# Synergistic Antimicrobial Action of Lactoferrin-Derived Peptides and Quorum Quenching Enzymes

**DOI:** 10.3390/ijms24043566

**Published:** 2023-02-10

**Authors:** Aysel Aslanli, Maksim Domnin, Nikolay Stepanov, Elena Efremenko

**Affiliations:** Chemical Faculty, Lomonosov Moscow State University, Lenin Hills 1/3, 119991 Moscow, Russia

**Keywords:** combined antimicrobials, lactoferrin, lactoferricin, antimicrobial activity, molecular docking, hexahistidine-containing organophosphorus hydrolase, hydrolysis, zearalenone

## Abstract

Combined use of various antimicrobial peptides (AMPs) with enzymes that hydrolyze the signaling molecules of the resistance mechanism of various microorganisms, quorum sensing (QS), to obtain effective antimicrobials is one of the leading approaches in solving the antimicrobial resistance problem. Our study investigates the lactoferrin-derived AMPs, lactoferricin (Lfcin), lactoferampin and Lf(1-11), as potential partners for combination with enzymes hydrolyzing lactone-containing QS molecules, the hexahistidine-containing organophosphorus hydrolase (His_6_-OPH) and penicillin acylase, to obtain effective antimicrobial agents with a scope of practical application. The possibility of the effective combination of selected AMPs and enzymes was first investigated in silico using molecular docking method. Based on the computationally obtained results, His_6_-OPH/Lfcin combination was selected as the most suitable for further research. The study of physical–chemical characteristics of His_6_-OPH/Lfcin combination revealed the stabilization of enzymatic activity. A notable increase in the catalytic efficiency of action of His_6_-OPH in combination with Lfcin in the hydrolysis of paraoxon, *N*-(3-oxo-dodecanoyl)-homoserine lactone and zearalenone used as substrates was established. Antimicrobial efficiency of His_6_-OPH/Lfcin combination was determined against various microorganisms (bacteria and yeasts) and its improvement was observed as compared to AMP without enzyme. Thus, our findings demonstrate that His_6_-OPH/Lfcin combination is a promising antimicrobial agent for practical application.

## 1. Introduction

The search for new antimicrobial agents to effectively suppress the growth and development of various microbial contaminants is one of the most important tasks today in medicine, sanitation, various fields of industry and agricultural production. The current modern approach to solving these problems is considered to be the combined use of various biologically active agents that have a different substrate spectrum of antimicrobial effects and exhibit a different mechanism of inhibition against microbial contaminants [1,2,3]. Such agents include antimicrobial peptides (AMPs), which are molecules produced by living organisms as a protective “shield” against various bacterial and fungal infections [4]. Recently, a few studies have revealed that bacteria can develop resistance to AMPs. However, in recent years, AMPs have attracted the attention of the world scientific community as an alternative to traditional antibiotics due to the wide range of antimicrobial actions against bacteria, mycelial fungi and yeast [5,6,7]. At the same time, the ability of several AMPs taken in combinations to delay the evolution of resistance in the pathogenic bacteria have been demonstrated [8,9].

AMPs obtained from food proteins, especially those with the status of GRAS (Generally recognized as safe), are of particular interest; they can be found among a large number of different molecules [10], including lactoferrin-derived AMPs. Lactoferrin itself is a serum iron-binding protein belonging to the transferrin family and is one of the most well-studied milk proteins exhibiting high antimicrobial, antiviral, anti-inflammatory, antioxidant and anti-cancer activities [11]. Lactoferrin is produced by epithelial cells of various organs and presents in most secretory fluids (0.001–6 g/L), including colostrum, milk, saliva, bile (including gastric and intestinal juice), urine and vaginal fluid [12]. As a result of careful studies conducted over many years of mainly antimicrobial properties and the mechanism of action of lactoferrin isolated from various sources (human, cow, buffalo, deer, ship, mouse etc.) [13,14,15,16], it was determined that both lactoferrin itself and its polypeptide derivatives (lactoferricin (Lfcin), lactoferampin (Lfampin) and the short peptide Lf(1-11)) of bovine origin in most cases exhibit significantly higher antimicrobial activity against various yeasts, Gram-positive (G(+)) and Gram-negative (G(−)) bacterial cells (Table 1). At the same time, the highest antimicrobial activity is noted for bovine Lfcin. A number of studies have demonstrated that combination of different antimicrobial agents with lactoferrin and its AMPs allows to obtain drugs with increased antibacterial, antifungal, antiviral or anticancer activity [17,18,19,20,21].

It is known that most G(−) bacteria, as well as yeast, use lactone-containing compounds as signaling molecules of the quorum sensing mechanism (QS), which provides cells in populations with high cell density with increased ability to survive by interacting with each other, and demonstrating improved cell resistance to antimicrobial agents. Among these auto-inductor molecules, various *N*-acyl homoserine lactones (AHLs), γ-butyrolactone and its derivatives are known [27,28]. Various enzymes, the so-called Quorum Quenching (QQ) catalysts, are used as one of the main ways to overcome the formation of QS and increase microbial cells’ resistance to the effects of antimicrobial substances. Such enzymes include lactonases and acylases capable of hydrolyzing lactone-containing QS signaling molecules [29,30]. The use of QQ enzymes in combination with clinical antibiotics is one of the most popular modern methods in antimicrobial therapy aimed at destabilizing microorganism cells by inhibiting their QS and restoring their sensitivity to the antibiotics used [31].

In this regard, from a scientific and practical point of view, the combination of lactoferrin-derived AMPs with enzymes that hydrolyze the signaling molecules of microbial QS for the potential production of effective antimicrobial drugs with the possibility of both external and possibly internal safe use is interesting.

It was previously shown that the combined use of QQ enzymes, such as penicillin acylase (PvdQ acylase) and hexahistidine-containing organophosphorus hydrolase (His_6_-OPH) exhibiting acylase and lactonase activity against various lactone-containing compounds, respectively, with various antibiotics and AMPs leads to an increase in the action effectiveness of the latter and an expansion in the spectrum of microbial objects for their exposure [32,33,34].

Taking this information into account, the purpose of this work was to study the possibility of combining these enzymes hydrolyzing lactone-containing QS signaling molecules with lactoferrin and its derivatives to obtain biologics with improved antimicrobial efficacy relative to the initial level of the AMPs themselves. For this purpose, models of the interaction of His_6_-OPH and PvdQ acylase with lactoferrin and its AMP derivatives were initially obtained using molecular modeling methods, and the interaction characteristics in the obtained models were studied. Further, it was decided to obtain and investigate the catalytic and physical–chemical characteristics of the most promising combinations of enzymes with AMPs, and to additionally assess a possible effect of enzymes combined with AMPs on their antimicrobial activity manifested against cells of various bacteria and yeast.

## 2. Results

### 2.1. Molecular Docking of Human and Bovine Lactoferrin-Derived AMPs to the Surface of Lactone Hydrolyzing Enzymes, His_6_-OPH and PvdQ Acylase

**Figure 1:**
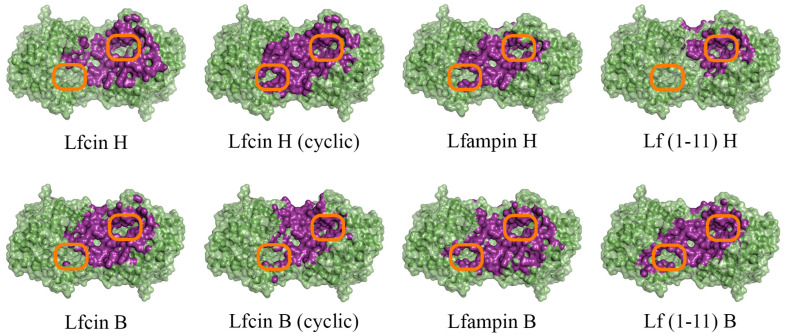
Front view of domains for binding of human (H) and bovine (B) lactoferrin-derived AMPs at pH 7.5 on the surface of His_6_-OPH homodimer (colored green). The atoms located within 4 Å of any AMP atom and the corresponding molecular surface are colored purple. The entrances to the active sites of His_6_-OPH dimer are highlighted with orange boxes.

To evaluate the intermolecular interactions of enzymes hydrolyzing lactone-containing molecules (His_6_-OPH and PvdQ acylase) with AMP derivatives of both bovine and human lactoferrin, the docking of Lfcin, Lfampin and Lf(1–11) molecules to the surface of the molecules of these two enzymes at pH 7.5, as the closest to physiological conditions, was carried out using the molecular modeling methods (Figure 1 and Figure S1).

**Figure 2:**
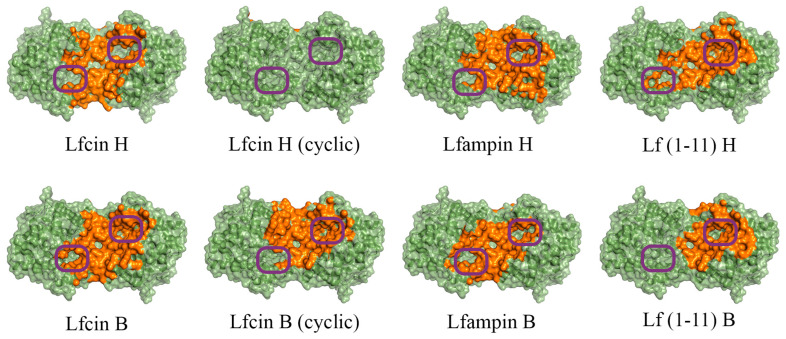
Front view of domains for binding of human (H) and bovine (B) lactoferrin-derived AMPs at pH 10.5 on the surface of His_6_-OPH homodimer (colored green). The atoms located within 4 Å of any AMP atom and the corresponding molecular surface are colored orange. The entrances to the active sites of His_6_-OPH dimer are highlighted with purple boxes.

In the case of His_6_-OPH, molecular docking was also performed at pH 10.5 (Figure 2), which corresponds to its maximum catalytic activity [35]. Since it is known that the Lfcin molecule can exist in both linear and cyclic forms, and exhibit equally high antimicrobial activity, both forms were used during molecular docking to the surfaces of both enzymes [36,37].

It was determined that the entrance to at least one active center on the surface of the dimeric form of the enzyme His_6_-OPH, in which it usually exists, remains available for the catalysis both at pH 7.5 (human Lfcin (linear), bovine Lfcin (linear and cyclic), human Lfampin and human Lf(1-11)) and at pH 10.5 (human Lfcin (linear and cyclic), bovine Lfcin(cyclic), human Lfampin and bovine Lf(1-11)) in the case of five molecules of AMPs from eight studied variants. At the same time, in the case of the cyclic form of human Lfc in, both active centers in the enzyme, dimer remained completely accessible for catalytic reactions at pH 10.5.

During the interaction of all variants of AMPs with the PvdQ acylase surface, a significant overlap of the active center of the enzyme was revealed (Figure S1).

To study the “enzyme:AMP” interaction characteristics in the obtained lactone hydrolyzing models, the values of following parameters were estimated: the affinity (interaction energy) of AMP molecules to the surface of His_6_-OPH and PvdQ acylase molecules, the area occupied by AMP molecules on the entire surface of each enzyme and near its active centers, the total surface charge of AMP molecules, as well as the distribution of charges on their surface (Table 2 and Table S1; Figures S2–S4).

Based on the obtained results, it was determined that the affinity of the molecules of bovine Lfcin to the surface of both His_6_-OPH and PvdQ acylase was significantly higher than for human Lfcin at both pH values used in the study (Table 2). At the same time, conversely, the affinity value for the human variant of this AMP turned out to be relatively higher than for the bovine variant in the case of Lfampin.

There was no noticeable difference in the obtained affinity values for bovine and human Lf(1-11) molecules. At the same time, the affinity of the AMP molecules to the surface of PvdQ acylase was relatively higher than to the surface of His_6_-OPH.

In the case of His_6_-OPH, the largest value of the affinity of the AMP molecule to the enzyme surface was noted for cyclic bovine Lfcin for both pH values (7.5 and pH 10.5), and the smallest values were determined for linear human Lfcin also at two pH values.

It should be noted that there were no patterns of changes in the affinity of AMP molecules to the His_6_-OPH surface during the transition between the two pH values. The charge of the surface of AMP molecules decreased with pH increasing, while the most significant decrease in charge was observed for human and bovine Lfcin molecules (2.5 and 2 times, respectively).

Analysis of the results of determining the size of the area occupied by AMP molecules on the surface of enzymes showed that there was a strong overlap of the active center of the PvdQ acylase by all AMP molecules (~0.7%).

In the case of interactions of AMP molecules with His_6_-OPH, in which at least one free active center remained in the dimer of the enzyme, AMPs occupied the smallest surface area near its active centers (0–0.1%). At the same time, the maximum surface area near the active centers of the enzyme occupied by AMP molecules was 0.3%. The values of the total area occupied by AMP molecules on the entire surface of His_6_-OPH varied in the range from 10 to 21%.

Thus, based on the analysis of the results obtained by molecular modeling, it was determined that the PvdQ acylase actually cannot exhibit catalytic activity in combination with the studied AMPs due to the blocking of the active center of the enzyme by the molecules of these polypeptides.

Taking this into account, the enzyme His_6_-OPH was selected as a QQ enzyme for experimental preparation of combinations with AMPs in the continuation of this study. Bovine Lfcin was chosen as the most optimal AMP for combination with His_6_-OPH, first based on the results of molecular docking, and second based on the literature data on this polypeptide [16], as such a lactoferrin derivative, which has the highest antimicrobial activity among its other AMP derivatives.

Since lactoferrin has similar antimicrobial activity to its AMP derivatives [15], it was also used in further wet experiments studying the catalytic and physical–chemical properties of combinations of the AMP with His_6_-OPH.

### 2.2. Catalytic and Physical–Chemical Characteristics of His_6_-OPH in Combination with Bovine Lactoferrin and Lactoferricin

**Figure 3:**
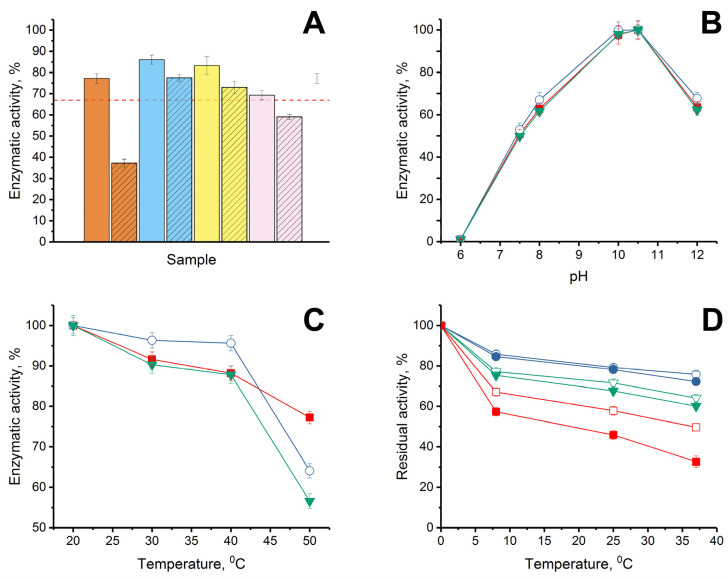
(**A**) Stability of enzymatic activity of His_6_-OPH without AMPs (dashed red line) and in combinations with lactoferrin (solid) and Lfcin (crosshatched), formed at pH 7.5 in the presence of 150 mM NaCl when the “enzyme:AMP” ratios were 1:0.5 (orange), 1:1 (blue), 1:2 (yellow) and 1:5 (pink) during exposure of the combinations for 72 h at 8 °C in 50 mM K-phosphate buffer (pH 7.5). Initial level of enzyme activity was assessed as 100%. (**B**) pH dependence of His_6_-OPH activity estimated in the absence (■) and presence (**◯**, **▼**) of lactoferrin and Lfcin, respectively. (**C**) Thermal inactivation of enzymatic activity of His_6_-OPH (■), His_6_-OPH/lactoferrin (**◯**), and His_6_-OPH/ Lfcin (**▼**) combinations, formed at pH 7.5 in the presence of 150 mM NaCl during exposure for 15 min at various temperatures. (**D**) Thermal stability of enzymatic activity of His_6_-OPH (□, ■), His_6_-OPH/lactoferrin (**◯**, **●**), and His_6_-OPH/ Lfcin (**▽**, **▼**) combinations, formed at pH 7.5 in the presence of 150 mM NaCl during exposure for 72 h at 8, 25 and 37 °C at a final protein concentration of 0.1 g/L (□, **◯**, **▽**) and 0.002 g/L (■, **●**, **▼**). The values are presented as the average values of the enzyme catalytic characteristics in the hydrolysis reactions of the three studied substrates (*N*-(3-oxo-dodecanoyl)-L-homoserine lactone, paraoxon and zearalenone).

Initially, the stability of the enzymatic activity of His_6_-OPH was investigated when it was combined with bovine AMP under conditions of varying the ratio “enzyme: AMP”. Such a study should have made it possible to determine the possibility of maintaining the maximum catalytic activity of the enzyme with the most favorable molecular ratio of the enzyme and AMP in the combinations created. For this purpose, combinations of His_6_-OPH with bovine lactoferrin or bovine Lfcin were experimentally obtained at different ratios “enzyme:AMP” (1:0.5, 1:1, 1:2 and 1:5). It should be noted that the 1:5 ratio for the combination was calculated based on the results of molecular docking, and the other variants of the ratios were used for research and verification of the results of computer modeling (Figure 3).

Regarding His_6_-OPH, considering that QQ-enzyme catalyzes the hydrolysis of various substrates and is able to successfully hydrolyze not only AHLs, but also organophosphorus pesticides and chemical warfare agents [38], as well as mycotoxins [39], the average value of residual activity (as a percentage of initial activity level) was estimated when the stability of the catalytic activity of the enzyme was controlled. The stability of the catalytic activity of His_6_-OPH during storage was determined in the hydrolytic reactions with three different substrates: organophosphate pesticide, paraoxon; signaling molecule-inducer of bacterial QS in *Pseudomonas* sp. cells, *N*-(3-oxo-dodecanoyl)-L-homoserine lactone; and lactone-containing mycotoxin, zearalenone (Figure 3A). When comparing the properties of His_6_-OPH in combinations with bovine lactoferrin and bovine Lfcin obtained at the same ratios, it was determined that combinations of the enzyme with both of the AMPs, which were obtained at a ratio of 1:1, were the most catalytically stable. In this regard, this ratio was further used in the estimations of the catalytic characteristics of the enzyme combinations with AMPs.

The pH dependence of the catalytic activity of His_6_-OPH in combinations with lactoferrin and Lfcin of bovine origin (Figure 3B) and the thermal inactivation of His_6_-OPH in the content of same combinations at different temperatures were studied in comparison with the initial enzyme without AMPs (Figure 3C). It was determined that neither lactoferrin itself nor bovine Lfcin as AMP derivative of lactoferrin had a negative effect on the catalytic activity of His_6_-OPH at different pH values, and the pH dependence of the enzyme in combination with AMPs remained the same as that of the original His_6_-OPH.

The study of the influence of dilution on the thermal stability of enzymatic activity of His_6_-OPH in complexes with AMPs at a constant salt concentration of 150 mM, which corresponds to a physiological salt concentration, showed that in combinations with AMPs, the thermostability of the enzymatic activity of His_6_-OPH was significantly higher compared to native enzyme even after dilution by 50 times (from 0.1 g/L to 0.002 g/L) (Figure 3D). For example, after 72 h of exposure at 37 °C, the residual enzymatic activity of His_6_-OPH in a solution with concentration of 0.1 g/L was 94–95% compared with a solution with concentration of 0.002 g/L in combinations with lactoferrin and Lfcin, whereas the residual enzymatic activity of His_6_-OPH alone was 65%.

When studying the thermal inactivation of the enzyme separately or as part of combinations in the temperature range from 20 to 40 °C, no negative changes in the enzymatic activity were detected. Moreover, in the presence of lactoferrin, there was an obvious increase in the catalytic activity of His_6_-OPH in the specified temperature range, which, of course, testified to the evident stabilization of the enzyme in this combination, and in addition created a clear attraction for the practical application of the enzyme in this particular variant, with increased stability, without changing the studied physical–chemical properties.

The catalytic characteristics of the enzyme were determined in the hydrolytic reactions of three different substrates (organophosphorus compound, paraoxon; bacterial signaling molecule-inducer of QS in *Pseudomonas* sp., *N*-(3-oxo-dodecanoyl)-L-homoserine lactone; and lactone-containing mycotoxin, zearalenone) in the presence and absence of lactoferrin or bovine Lfcin to study the possible inhibitory effect of these AMPs on the enzymatic activity of His_6_-OPH, (Table 3, Figure S5).

As a result, it was determined that in the presence of both lactoferrin and bovine Lfcin, there was a decrease in the value of the Michaelis constant for His_6_-OPH and, consequently, an increase in the efficiency constant of the catalytic action of the enzyme. The maximum of the efficiency constant was noted for the combination of His_6_–OPH/bovine Lfcin for all the studied substrates. This phenomenon may be associated with different localization of bovine lactoferrin or Lfcin molecules on the surface of the dimer enzyme molecule, which may lead to some changes in the conformation of the His_6_-OPH molecule and the spatial conformation state of the active center of the enzyme, thereby affecting the binding rate of the substrate. Similar changes in the catalytic characteristics of His_6_-OPH were previously observed with some other polypeptides [40].

To study the changes in the particle sizes of His_6_-OPH when combined with bovine lactoferrin and Lfcin, the effective hydrodynamic diameters (Deff) of nanoparticles in solutions with His_6_-OPH, His_6_-OPH/Lactoferrin and His_6_-OPH/Lfcin (Figures S6 and S7) were defined. It was determined that the main Deff in solutions with His_6_-OPH, His_6_-OPH/Lactoferrin and His_6_-OPH/Lfcin is 6.5 ± 0.9, 32.7 ± 5.1 and 12.1 ± 1.3 nm, respectively. Thus, it was established that the largest particle size was observed for the combination of His_6_-OPH/Lactoferrin, whereas the particle size of His_6_-OPH in combination with Lfcin was 1.6 times higher compared to the enzyme itself. These data confirmed the possible formation of non-covalent complexes of His_6_-OPH with AMPs.

### 2.3. Estimation of Antimicrobial Characteristics of Bovine Lactoferrin and Lactoferricin against Different Microorganisms in the Presence and in the Absence of His_6_-OPH

**Figure 4:**
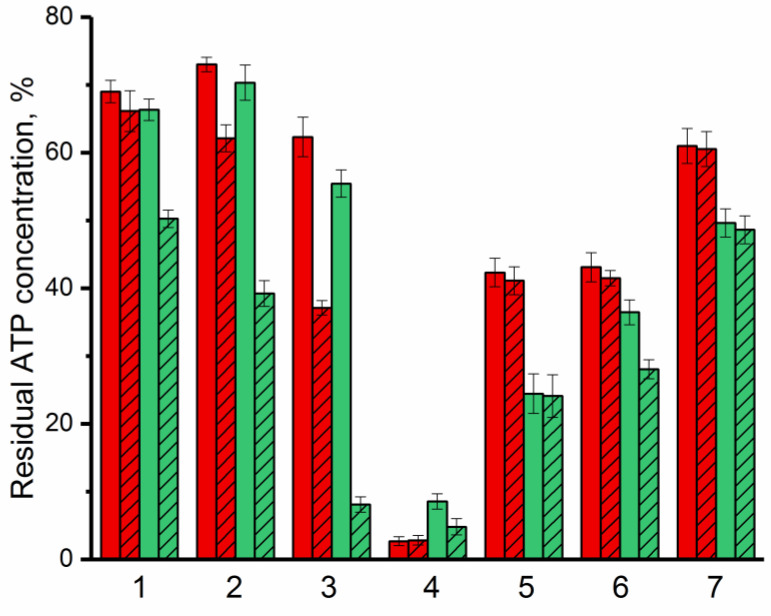
Residual ATP concentration in cells as a manifestation of antimicrobial activity of lactoferrin (red) and Lfcin (green) against G(−) (*Pseudomonas putida* (1), *Pseudomonas* sp. (2), *Agrobacterium tumefaciens* (3)) and G(+) (*Bacillus subtilis* (4), *Lactobacillus* sp. (5)) bacteria, and yeasts (*Saccharomyces cerevisiae* (6), *Candida spp.* (7)) in the absence (solid) or in the presence of His_6_-OPH (crosshatched). Concentration of cells in the medium without any additives was assessed as 100%.

The antimicrobial activity of bovine lactoferrin and Lfcin against G(−) (*Pseudomonas putida* B4589, *Pseudomonas* sp. 78G, *Agrobacterium timefaciens* B-8833) and G(+) (*Bacillus subtilis* B522, *Lactobacillus* sp. B3730) bacterial cells and yeast (*Saccharomyces cerevisiae* strain Vintage white (Enartis Ferm, San Marino, Italy) and *Candida* sp. Y94) was investigated to estimate the effect of combining lactoferrin and Lfcin with enzyme on the antimicrobial efficacy of these AMPs (Figure 4). To determine antimicrobial activity of AMPs, the bioluminescent ATP-metry was used as a new and modern method that allows assessing the physiological state of bacterial cells and tracking changes in their metabolic activity [41]. To monitor changes in the number of bacterial cells, CFU-ATP calibration curves were used (Figure S9).

It was established that with respect to all studied microorganisms, except *Bacillus subtilis*, bovine Lfcin acts comparatively better than lactoferrin itself. The maximum antimicrobial activity of both bovine lactoferrin and Lfcin was noted against G(+) bacterial cells. In many of the cases considered, the presence of the enzyme in an environment with antimicrobial agents led to an increase in their effectiveness against the studied microorganisms. At the same time, the most noticeable synergistic effect of AMP and enzyme was noted in combination of His_6_-OPH with bovine Lfcin. β-Lactam antibiotic ampicillin with a concentration ~30 times higher compared to the concentrations of available AMPs was used as a positive control. The decrease in intracellular ATP concentrations in the presence of ampicillin was 20% and 28% in the case of G(−) and G(+) bacterial cells, respectively, which was ~3.5 times lower than in the case of lactoferrin or Lfcin.

## 3. Discussion

Due to the high antimicrobial activity and wide spectrum of action, bovine lactoferrin-derived AMPs, particularly Lfcin, which can be obtained by hydrolysis from lactoferrin, are of particular interest for the preparation of combined antimicrobials. A notable fact about these AMPs (Table 1) is that they are capable of showing high antimicrobial activity against various microorganisms, whereas the whole protein lactoferrin is required for the manifestation of antiviral activity [42]. Since it has recently been demonstrated that bacteria can develop resistance to AMPs and that their combined use allows to delay the evolution of pathogen resistance [8,9], the use of combined forms of lactoferrin-derived AMPs seems to be the most appropriate way to avoid the development of resistance. Such combinations can be based not only on AMPs or antibiotics [43,44], but also on enzymes that are active against different G(+) and G(−) bacteria [45,46,47]. In vitro studies of the combined use of various AMPs derived from human and bovine lactoferrin with traditional antibiotics revealed the presence of a synergistic effect between polypeptides and antibiotics; however, the obtained results were insufficient to produce therapeutic effects in vivo [48]. In this regard, the search for new solutions among possible combinations of AMPs from lactoferrin continues.

Combination of QQ enzymes with antimicrobial agents is one of the most current and effective solutions in antimicrobial therapy. For example, it has been demonstrated that the use of YtnP lactonase in combination with antibiotics leads in an increase in its efficacy in the treatment of systemic *Pseudomonas aeruginosa* infections [31,49]. The interest in conducting this study was based on the desire of the authors to explore the possibility of combining lactoferrin-derived AMPs with lactone-hydrolyzing enzymes, His_6_-OPH and PvdQ acylase to obtain antimicrobials with improved action against various microorganisms by combining AMPs with QQ-enzymes.

Using the molecular docking method (Table 2), it was shown that the most suitable partners for combination are His_6_-OPH as a lactone-hydrolyzing enzyme and bovine Lfcin as a lactoferrin-derived peptide, since it is known that negatively charged amino acid residues are mainly located in the region of the active centers of the enzyme [38]. Among the considered variants of AMPs, it was bovine Lfcin the molecular surface of which had the highest positive charge and, therefore, it was initially expected to have a maximum interaction with the His_6_-OPH dimer in the region of its active centers and their possible overlap. However, it is interesting that the results of molecular modeling showed that despite the location of bovine Lfcin molecules on the anterior surface of the enzyme dimer, the area of the location of active centers remains accessible for substrates and, accordingly, for the course of catalytic reactions. These results are consistent with the results we obtained earlier with some other positively charged AMPs [33], which demonstrated unpredictable connections with enzymes, but these expectations were based on the well-known theory of inter-charge interactions. At the same time, the highest interaction energy with the surface of the His_6_-OPH dimer was also observed with the bovine Lfcin molecule.

A study of the catalytic and physical–chemical characteristics of the enzymatic activity of His_6_-OPH in combination with bovine Lfcin showed that the efficiency of the catalytic reaction increases with respect to several known substrates of this enzyme (pesticide, AHL and mycotoxin) (Table 3). The obtained results indicated that not only does the combination of His_6_-OPH with bovine Lfcin not have a negative effect on the enzymatic properties, but, on the contrary, it allows obtaining an enzymatic biologic with improved catalytic characteristics. Thus, attempting to improve the properties of the AMPs, we suddenly obtained more stable and active enzyme with “antimicrobial” function.

When studying the antimicrobial activity of bovine Lfcin in the presence of an enzyme, an increase in the effectiveness of the AMP was detected not only against G(−) bacterial cells, which use lactone-containing signaling molecules that can be hydrolyzed by His_6_-OPH [50], but also against yeast cells. These results are consistent with the data we previously obtained for combinations of the enzyme with bacitracin [32] and confirm the previously stated idea that His_6_-OPH obviously possesses the hydrolytic activity in relation to lactone-containing signaling molecules (γ-butyrolactone and its derivatives) of yeasts involved in the QS development.

Another noteworthy result of this study is that, along with the maximum antimicrobial activity of the combination of His_6_-OPH/bovine Lfcin, manifested in relation to G(+) cells of *Bacillus subtilis*, the lowest antimicrobial activity of this combination was noted in relation to *Lactobacillus* sp. cells. It is interesting that 10 years ago, the data obtained in the work that studied antimicrobial action of bovine lactoferrin and its AMP derivatives in the media with lactoferrin-resistant probiotic cells allowing prevention of foodborne pathogens were published [51,52]. In connection to this, we added QQ-enzyme in combination with the same AMPs and confirmed the results. This is undoubtedly a positive fact, enabling the possible joint use of a combination of His_6_-OPH/bovine Lfcin and probiotic cells of *Lactobacillus* sp. as possible additives, for example, in the feed of farm animals, birds, etc.

The introduction of such a combination as an additive can potentially provide multiple positive effects simultaneously: detoxification of mycotoxin and pesticides, suppressive effect on yeast contaminants in the feed and increase in effectiveness of feed conservation with minimal impact on probiotics often introduced into the feed. If we also take into account that lactoferrin itself is capable of inhibiting the cytotoxicity effect induced by highly toxic lactone-free mycotoxins, in particular aflatoxin B1 and aflatoxin M1 [53], then, in general, we can expect a combined detoxifying effect on various mycotoxins due to the action of both enzyme His_6_-OPH and AMP. The enzyme catalyzes the destruction of some mycotoxins [54,55], whereas the AMPs work as antioxidants reducing the amounts of oxygen active forms generated in response to appearance of mycotoxins. As an AMP produced in living organisms, Lfcin has a relatively mild and selective mechanism of action against microorganism compared to many other AMPs. In this regard, the His_6_-OPH/Lfcin combination has great potential for use as a biologically active additive, e.g., in various cosmetic products, most of which are known to contain different peptides [56] or in the different wound healing materials, particularly infected foot wounds in patients with diabetes [57]. The His_6_-OPH/Lfcin combination can also be used to treat and softly reduce contamination of a variety of surfaces, such as fruits and vegetables, to prevent the development of pathogens and extend their shelf life.

Early preclinical studies have shown that bovine lactoferrin is well tolerated by newborns and retains biological activity in the gut, and that administering bovine lactoferrin to very low birthweight infants protects against late sepsis and necrotizing enterocolitis resulting from various infections [58]. Moreover, a recent study of the impact of oral administration of lactoferrin saturated with manganese on animal model of neonatal sepsis induced by bacterial translocation showed a significant increase in the *Lactobacillus* bacterial population, which contributed to the strengthening of the intestinal barrier and inhibition of the phenomenon of translocation [59]. This fact allows us to assume that the His_6_-OPH/Lfcin combination has the potential to be used as an additive with antimicrobial activity to infant milk formula or baby dairy products, especially considering the fact that His_6_-OPH has been previously successfully applied in vivo as an antidote and its non-toxicity has already been investigated and confirmed [38,55].

Lactoferrin demonstrates high affinity to its receptors overexpressed on cancer cells, which facilitates a great potential of lactoferrin nanoparticles for active targeting of tumor and overcoming such physiological barriers as the gastrointestinal barrier and blood–brain barrier. Thereby, lactoferrin nanoparticles are actively used as a carrier for the drug delivery to the central nervous system [60]. Recently, the potential role of lactoferrin in the treatment of neurodegenerative diseases, particularly Alzheimer’s disease (AD), has also been investigated, and it has been shown that the administration of lactoferrin significantly improves cognitive functions and increases the serum levels of acetylcholine, serotonin, antioxidant, and anti-inflammatory markers [61]. Taking into account the fact that the molecules of various organophosphorus compounds could interact with decarboxylases, as we have established earlier in silico, leading to the inhibition of these enzymes and to the development of a number of neurodegenerative diseases [62], it can be assumed that the His_6_-OPH/Lfcin combination could be used as a potential antidote in the prevention of the development of neurodegenerative diseases.

Therefore, the various ways of application of the combinations developed in this research may become the topic of a new separate study, not only for the authors, but also readers of the present paper.

## 4. Materials and Methods

### 4.1. Materials

Lactoferrin samples derived from bovine colostrum, porcine pepsin, paraoxon, N-(3-oxo-dodecanoyl)-L-homoserine lactone, zearalenone, ampicillin, Coomassie Brilliant Blue R-250 and Coomassie Brilliant Blue G-250 were purchased from Sigma-Aldrich (Darmstadt, Germany). Standard ATP reagent was purchased from Lyumtek OOO (Moscow, Russia).

### 4.2. Computational Methods

The structure of His_6_-OPH dimer was prepared using the known crystallographic structure of OPH (PDB: 1QW7) which was modified by His_6_-tag as described previously [33].

Crystallographic structure of penicillin acylase PvdQ from *Pseudomonas aeruginosa* was obtained from the Protein Data Bank (PDB 4m1j).

ChemBioDraw software (ver. 12.0, CambridgeSoft, Waltham, MA, USA) and ChemBio3D were used to create AMPs structures and to apply energy minimization with force field MM2.

Adaptive Poisson–Boltzmann solver (APBS) and PDB2PQR servers (ver. 1.4.2.1 and 2.1.1, respectively, available at http://www.poissonboltzmann.org/, accessed on 15 September 2022) with PARSE forcefield and default settings were used to calculate the surface charge distribution of His_6_-OPH, PvdQ acylase, Lactoferrin and AMPs [63]. Structures obtained in PQR and PDB format were converted to the PDBQT format using AutoDockTools (as part of MGLTools ver. 1.5.6, available at http://mgltools.scripps.edu/, accessed on 15 September 2022) with atomic charges calculated with the Gasteiger–Marsili method [64].

AutoDockVina (ver. 1.1.2, available at http://vina.scripps.edu/, accessed on 15 September 2022), which includes several terms in its own scoring function (Gaussian, repulsion, hydrophobic, hydrogen bonding, and the number of rotatable bonds), was used for docking [65]. Conformations of AMPs at pH 7.5 and 10.5 were docked to the surface of corresponding conformations of enzymes obtained at the same pH values. This was performed on a desktop computer equipped with Intel Pentium Dual-Core CPU E5400 2.7 GHz and 3 GB of available memory. Briefly, the grid box was approximately centered on the center of mass of the enzyme molecule. The size of the grid box was chosen so that the enzyme surface was within the box with an additional margin. Calculations were performed with default program options. Based on our previous studies with His_6_-OPH and PvdQ acylase, and results obtained in the development of new effective antibacterials [33,54], the 12 best poses with minimal energy were selected. The “*get_area*” function of PyMOL was used to calculate solvent accessible area occupied by AMPs on the surface of enzymes.

### 4.3. Hydrolysis of Bovine Lactoferrin to Lactoferricin and Its Purification

Lactoferricin was purified using the method of Tomita et al. [66] involving porcine pepsin (Sigma-Aldrich, Darmstadt, Germany). Lactoferrin derived from bovine colostrum (Sigma-Aldrich, Darmstadt, Germany) was dissolved in distilled water at a concentration of 10%, and the pH was adjusted to 2.5. Porcine pepsin was added to a final concentration of 0.3%. The hydrolysis reaction was performed at 37 °C for 5 h and terminated by heating at 80 °C for 15 min. The reaction mixture containing pepsin was neutralized by addition of NaOH, and the pH was adjusted to 7.0. The precipitate of insoluble peptides formed in the reaction mixture was removed by centrifugation at 11,000 g for 0.5 h. Obtained supernatant was fractionated by ion-exchange chromatography according to the previously described procedure [67] with slight modifications in order to purify Lfcin.

Lactoferrin hydrolysate was thawed at 8 °C, applied to 5.0 mL of cation exchange column and fractionated by 50 mM phosphate buffer (pH 7.5) containing 2.0 M NaCl at a flow rate of 2 mL/min. The fractionation process was monitored by measuring the absorbance of the eluent at 280 nm. The fractionated peptides were identified using FindPept software [68] and analysed by SDS-PAGE in a 12% polyacrylamide gel using a Miniprotean II cell (Bio-Rad, Hercules, CA, USA) followed by Coomassie Blue (R-250) (Sigma-Aldrich, Darmstadt, Germany) staining (Figure S8). The resulting fractions were stored at −35 °C.

### 4.4. Preparation of the Enzyme and Its Combinations with AMPs with Their Further Characterization

His_6_-OPH was isolated from biomass of developed *E. coli* SG13009[pREP4] strain, purified and characterized by previously published methods [69]. In short, protein concentration was determined by Bradford assay with Coomassie Brilliant Blue G-250 (Sigma-Aldrich, Darmstadt, Germany) and its purity was analyzed by sodium dodecyl sulfate polyacrylamide gel electrophoresis in 12% polyacrylamide gel using Mini-PROTEAN II cell (Bio-Rad, Hercules, CA, USA) followed by Coomassie Brilliant Blue R-250 (Sigma-Aldrich, Germany) staining. Enzymatic activity was measured using the Agilent 8453 UV–visible spectroscopy system (Agilent Technology, Waldbronn, Germany) equipped with a thermostatted analytical cell with 10 mM aqueous Paraoxon (Sigma-Aldrich, Darmstadt, Germany) solution at 405 nm in a 100 mM Na-carbonate buffer (pH 10.5). Concentration of His_6_-OPH was ca. 2.5 nM in the reaction cuvette. One unit of enzyme activity (U) was defined as the quantity of the enzyme necessary to hydrolyze 1 μmol of Paraoxon per min at 25 °C. The purity and specific activity of His_6_-OPH preparation obtained (*M*_W_ ≈ 37 kDa) was ca. 98% and 6000 U/mg.

Combinations of antimicrobial agents (bovine Lactoferrin and Lfcin) with His_6_-OPH were prepared according to the following procedure: solution of antimicrobial agent at concentration of 0.2 g/L in 50 mM K-phosphate buffer (pH 7.5) containing 150 mM NaCl was mixed with solution of 0.2 g/L His_6_-OPH in the same buffer at 1:1 (*v*/*v*) ratio and exposed for 30 min at room temperature. After that, organophosphate and lactonase activity of the combination prepared was measured as described earlier while adding varying Paraoxon, N-(3-oxo-dodecanoyl)-L-homoserine lactone or zearalenone concentrations in a reaction mixture to determine catalytic characteristics [38,49].

The stability of enzymatic activity was studied with different ratios of His_6_-OPH:AMP. For that, solutions of enzyme and antimicrobial agent (Lactoferrin or Lfcin) were mixed with the final ratios of 1:0.5, 1:1, 1:2, 1:5 and exposed for 72 h at 8 °C. Residual activity was measured.

The effect of physical–chemical parameters on enzyme activity was tested. Kinetics of thermoinactivation were studied at 20–50 °C and pH 7.5 for 15 min and then cooled; residual activity was measured. Thermal stability of enzymatic activity was studied at 8, 25 and 37 °C after 72 h of exposition in 50 mM K-phosphate buffer (pH 7.5) containing 150 mM NaCl at final protein concentrations of 0.1 g/L and 0.002 g/L. Dependence of enzyme catalytic activity on the pH was determined using buffers with overlapping pH values: 50 mM phosphate buffer (pH 6.0; 7.5), 50 mM phosphate/100 mM carbonate buffer (pH 8.0) and 100 mM carbonate buffer (pH 10.0; 12.0).

The values of the Michaelis constant (*K*_m_) and the maximum rate of enzymatic reaction (*V*_max_) were calculated by hyperbolic approximation using the least squares method in Origin Pro (ver. 8.1 SR3, OriginLab, Northampton, MA, USA). The obtained *K*_m_ and *V*_max_ values were further used to calculate catalytic constant (*V*_max_/*E*_0_) and action efficiency constant (*V*_max_/(*E*_0_ × *K*_m_) values for enzymatic activity.

Effective hydrodynamic diameters (Deff) of nanoparticles were determined at 25 °C by DLS using a Zetasizer Nano ZS (Malvern Instruments Ltd., UK). Hydrodynamic size of nanoparticles was determined as described previously [70] by PTA using a NanoSight NS500 instrument (Malvern Panalytical, Malvern, UK) equipped with an 80 mW 532 nm laser. The size distribution of nanoparticles was calculated using NanoSight software (ver. 2.3, Malvern Panalytical) on the basis of 5 independent experiments.

### 4.5. Determination of Antimicrobial Activity of Bovine Lactoferrin and Lactoferricin with or without His_6_-OPH

Antimicrobial efficiency of action of bovine lactoferrin or Lfcin alone or in combination with His_6_-OPH (1 g/L) was determined as described previously [33] with minor modifications. For that, overnight bacterial cultures (*Pseudomonas putida* B4589, *Pseudomonas* sp. 78G, *Agrobacterium tumefaciens* B-8833, *Bacillus subtilis* B522, and *Lactobacillus* sp. B3730) and yeasts (*Candida* sp., *Saccharomyces cerevisiae*) were used.

To accumulate biomass, the cells of bacteria were aerobically cultivated in the corresponding culture medium as described previously [33,49]. Yeast cells were grown in the corresponding culture medium as it was described previously [31]. The bacterial and yeast cells were cultivated using a thermostatically controlled Adolf Kuhner AG shaker (Basel, Switzerland) at 28 °C for G(−) and yeasts and 30 °C for G(+), with constant stirring at 150 rpm.

Cell growth was monitored with an Agilent UV-8453 spectrophotometer (Agilent Technology, Waldbronn, Germany) at 540 nm.

All microorganisms were separated from the nutrition media after cultivation by centrifugation. Further, the cells were suspended in a saline medium (ca. (1 ± 0.1) × 10^5^ cells/mL) prepared on the basis of 50 mM phosphate buffer (pH 7.5). The cells were exposed at room temperature for 4 h with Lactoferrin or Lfcin added in a concentration of up to 32 mg/L. β-Lactam antibiotic ampicillin was added in a concentration of up to 1 g/L and used as a positive control in antibacterial activity tests.

The concentration of intracellular ATP was determined using a standard ATP reagent (Lyumtek OOO, Moscow, Russia) and luciferin-luciferase method to evaluate the residual concentration of viable cells in exposed samples using published procedure [41]. The intensity of bioluminescence was recorded using a Microluminometer 3560 (New Horizons Diagnostic, Arbutus, MD, USA).

All data are presented as means of at least three independent experiments ± standard deviation (±SD). Statistical analysis was realized using SigmaPlot (ver. 12.5, Systat Software Inc., San Jose, CA, USA).

## 5. Conclusions

In this study, we demonstrated, for the first time, the possibility of combining of QQ enzyme His_6_-OPH with milk-derived AMP Lfcin in order to obtain combinations characterized by antimicrobial activity and a wide substrate spectrum of enzymatic activity. Combinations based on His_6_-OPH and bovine Lfcin appeared to be active both against various lactone-containing QS signaling molecules and substrate toxins of the enzyme. Since in the recent years lactoferrin and its derivative AMPs have attracted great interest from the scientific community due to their unique high antimicrobial, anti-cancer, anti-oxidant, etc., properties, His_6_-OPH/Lfcin combination has a great application potential for both external and internal use. Taking into account the fact that His_6_-OPH has been successfully applied in vivo as an antidote, especially in combination with poly(amino acid)-based polymers [37,54], and Lfcin is an AMP with GRAS status, the combination of His_6_-OPH/bovine Lfcin may be potentially used both as a biologically active additive to different baby dairy products, surface treatment products, cosmetics, wound healing materials and as an antidote against brain pathogens and organophosphorus compounds for the prevention of the development of such neurodegenerative diseases as AD, Parkinson’s disease, etc.

## Figures and Tables

**Table 1:** Characteristics of lactoferrin-derived AMPs.

AMPs [Reference]	Amino Acid Sequence of AMPs	Target of Action [MIC *, µg/mL]
Human Lfcin (1–11;17–41) [22,23,24,25]	GRRRSVQWCAVTKCFQWQRNMRKVRGPPVSCIKRDS	G(+) bacteria G(−) bacteria [>200]
Bovine Lfcin (17–41); β-sheets [22,23,24,25]	FKCRRWQWRMKKLGAPSITCVRRAF	G(+) bacteria [0.3–500] G(−) bacteria [1.6–>1000] Yeasts [0.31–400]
Human Lfampin (268–284) [24,25,26]	WNLLRQAQEKFGKDKSP	G(+) bacteria [4.3–20] ** G(−) bacteria [5.8–25] ** Candida albicans [0.7–2.1] **
Bovine Lfampin (268–284) [24,25,26]	WKLLSKAQEKFGKNKSR	
Human Lf(1–11) [24,25]	GRRRSVQWCAV	G(+) bacteria [1.6–6.3] G(−) bacteria [6.3–12.5] *Candida* sp. [>12.5]
Bovine Lf(1–11) [17,18]	APRKNVRWCTI	

* MIC is a minimal inhibiting concentration; ** LC_50_ is a 50% lethal concentration (µM).

**Table 2:** Values of occupied area and affinity of human (H) and bovine (B) lactoferrin-derived AMPs to the surface of His_6_-OPH dimer and PvdQ acylase.

AMP	Enzyme	pH	Area, %		Affinity, (kJ × mol^−1^)		
			Near Active Sites	Total	Mean	Median	Range
Lfcin H * (linear)	His_6_-OPH	7.5	0.1	10.3	−10.9	−11.1 ± 0.6	−10.4 (−11.4)
		10.5	0.1	21.8	−13.0	−13.7 ± 1.1	−12.2 (−13.7)
	PvdQ acylase	7.5	0.7	13.0	−19.9	−19.9 ± 0.2	−19.7 (−20.1)
Lfcin H (cyclic)	His_6_-OPH	7.5	0.3	12.3	−15.2	−15.1 ± 0.7	−14.6 (−15.9)
		10.5	0	14.1	−13.8	−13.8 ± 0.6	−13.4 (−14.2)
	PvdQ acylase	7.5	0.7	13.8	−24.2	−24.3 ± 0.3	−23.8 (−24.4)
Lfcin B * (linear)	His_6_-OPH	7.5	0.1	16.1	−22.5	−22.2 ± 1.1	−21.4 (−23.3)
		10.5	0.3	18.2	−21.0	−20.9 ± 1.1	−19.8 (−22.1)
	PvdQ acylase	7.5	0.6	14.7	−24.5	−24.3 ± 0.9	−23.8 (−25.0)
Lfcin B (cyclic)	His_6_-OPH	7.5	0.2	18.3	−27.6	−26.8 ± 2.1	−25.6 (−30.0)
		10.5	0.2	19.7	−26.1	−27.6 ± 2.1	−24.7 (−27.6)
	PvdQ acylase	7.5	0.7	10.5	−31.5	−31.4 ± 0.4	−31.4 (−31.8)
Lfampin H	His_6_-OPH	7.5	0.1	13.3	−22.6	−22.4 ± 0.8	−21.9 (−23.3)
		10.5	0.1	14.9	−24.6	−24.7 ± 0.9	−24.0 (−24.7)
	PvdQ acylase	7.5	0.7	12.6	−30.4	−30.1 ± 0.5	−30.1 (−30.9)
Lfampin B	His_6_-OPH	7.5	0.2	17.9	−18.9	−18.6 ± 1.0	−18.1 (−19.6)
		10.5	0.1	19.5	−19.8	−19.5 ± 0.9	−18.9 (−20.8)
	PvdQ acylase	7.5	0.7	11.7	−27.5	−27.6 ± 0.5	−27.2 (−28.0)
Lf(1-11) H	His_6_-OPH	7.5	0.2	10.6	−25.8	−25.7 ± 0.8	−25.1 (−26.4)
		10.5	0.4	12.6	−27.5	−27.4 ± 1.1	−26.8 (−28.0)
	PvdQ acylase	7.5	0.7	8.3	−25.5	−24.9 ± 1,4	−24.4 (−26.6)
Lf(1-11) B	His_6_-OPH	7.5	0.3	17.1	−27.8	−27.4 ± 1.0	−26.9 (−28.9)
		10.5	0.1	12.9	−25.3	−25.1 ± 1.0	−24.7 (−25.5)
	PvdQ acylase	7.5	0.7	9.6	−27.1	−27.2 ± 0.6	−26.8 (−27.2)

* Human (H) and bovine (B) lactoferrin-derived AMPs.

**Table 3:** Catalytic characteristics of His_6_-OPH in combination with bovine lactoferrin and Lfcin *.

Enzyme or Complex	*K*_m_ (μM)	*V*_max_ × E_0_^−1^ (s^−1^)	*V*_max_ ·× *E*_0_^−1^ × *K*_m_ ^−1^ (10^3^ s^−1^·M^−1^)
Substrate—Paraoxon			
His_6_-OPH	10.5 ± 2	5040 ± 140	(480 ± 105) × 10^3^
His_6_-OPH/Lactoferrin	6 ± 1	3270 ± 190	(545 ± 123) × 10^3^
His_6_-OPH/Lfcin	9 ± 1	5590 ± 120	(621 ± 82) × 10^3^
Substrate—*N*-(-3-oxo-dodecanoyl)-homoserine lactone			
His_6_-OPH	101 ± 7	1.8 ± 0.05	17.8 ± 1.7
His_6_-OPH/Lactoferrin	68 ± 4	1.2 ± 0.02	17.6 ± 1.3
His_6_-OPH/Lfcin	82 ± 6	2.9 ± 0.04	35.4 ± 3.1
Substrate—Zearalenon			
His_6_-OPH	3400 ± 97	0.15 ± 0.01	0.044 ± 0.004
His_6_-OPH/Lactoferrin	2000 ± 67	0.12 ± 0.01	0.058 ± 0.007
His_6_-OPH/Lfcin	2500 ± 82	0.16 ± 0.02	0.065 ± 0.01

* The values are presented as the average values of the enzyme catalytic characteristics in the hydrolysis reactions of the three studied substrates.

## Data Availability

Not applicable.

## Supplementary Materials

The supporting information can be downloaded at: https://www.mdpi.com/article/10.3390/ijms24043566/s1. References [63,71,72] are cited in supplementary materials.

## References

[1] Larsson D.G., Flach C.F. (2022). Antibiotic resistance in the environment. Nat. Rev. Microbiol..

[2] Vestergaard M., Frees D., Ingmer H. (2019). Antibiotic Resistance and the MRSA Problem. Microbiol. Spectr..

[3] Jamrozik E., Heriot G.S. (2022). Ethics and antibiotic resistance. Br. Med. Bull..

[4] Büyükkiraz E.M., Kesmen Z. (2022). Antimicrobial peptides (AMPs): A promising class of antimicrobial compounds. J. Appl. Microbiol..

[5] Talapko J., Meštrović T., Juzbašić M., Tomas M., Erić S., Horvat Aleksijević L., Bekić S., Schwarz D., Matić S., Neuberg M. (2019). The antimicrobial peptides and their potential clinical applications. Am. J. Transl. Res..

[6] Zhang C., Yang M. (2022). Antimicrobial peptides: From design to clinical application. Antibiotics.

[7] Spohn R., Daruka L., Lázár V., Martins A., Vidovics F., Grézal G., Méhi O., Kintses B., Számel M., Jangir P.K. (2019). Integrated evolutionaryanalysis reveals antimicrobial peptides with limited resistance. Nat. Commun..

[8] Maron B., Rolff J., Friedman J., Hayouka Z. (2022). Antimicrobial peptide combination can hinder resistance evolution. Microbiol. Spectr..

[9] López-García G., Dublan-García O., Arizmendi-Cotero D., Gómez Oliván L.M. (2022). Antioxidant and antimicrobial peptides derived from food proteins. Molecules.

[10] Bielecka M., Cichosz G., Czeczot H. (2021). Antioxidant, antimicrobial and anticarcinogenic activities of bovine milk proteins and their hydrolysates-A review. Int. Dairy J..

[11] Rosa L., Cutone A., Lepanto M.S., Paesano R., Valenti P. (2017). Lactoferrin: A natural glycoprotein involved in iron and inflammatory homeostasis. Int. J. Mol. Sci..

[12] Wang Y., Morton J.D., Bekhit A.E.D.A., Carne A., Mason S.L. (2021). Amino acid sequences of lactoferrin from red deer (Cervus elaphus) milk and antimicrobial activity of its derived peptides lactoferricin and lactoferrampin. Foods.

[13] Kell D.B., Heyden E.L., Pretorius E. (2020). The biology of lactoferrin, an iron-binding protein that can help defend against viruses and bacteria. Front. Immunol..

[14] Gruden Š., Poklar Ulrih N. (2021). Diverse mechanisms of antimicrobial activities of lactoferrins, lactoferricins, and other lactoferrin-derived peptides. Int. J. Mol. Sci..

[15] Ianiro G., Rosa L., Bonaccorsi di Patti M.C., Valenti P., Musci G., Cutone A. (2022). Lactoferrin: From the structure to the functional orchestration of iron homeostasis. BioMetals.

[16] Barakat A., Al-Majid A.M., Lotfy G., Ali M., Mostafa A., Elshaier Y.A. (2021). Drug repurposing of lactoferrin combination in a nanodrug delivery system to combat severe acute respiratory syndrome coronavirus-2 infection. Dr. Sulaiman Al Habib Med. J..

[17] Ramírez-Sánchez D.A., Arredondo-Beltrán I.G., Canizalez-Roman A., Flores-Villaseñor H., Nazmi K., Bolscher J.G., León-Sicairos N. (2021). Bovine lactoferrin and lactoferrin peptides affect endometrial and cervical cancer cell lines. Biochem. Cell Biol..

[18] Fernandes K.E., Payne R.J., Carter D.A. (2020). Lactoferrin-derived peptide lactofungin is potently synergistic with amphotericin B. Antimicrob. Agents Chemother..

[19] Małaczewska J., Kaczorek-Łukowska E., Wójcik R., Siwicki A.K. (2019). Antiviral effects of nisin, lysozyme, lactoferrin and their mixtures against bovine viral diarrhoea virus. BMC Vet. Res..

[20] Mahdi L., Mahdi N., Musafer H., Al-Joofy I., Essa R., Zwain L., Salmana I., Mater H., Al-Alak S., Al-Oqaili R. (2018). Treatment strategy by lactoperoxidase and lactoferrin combination: Immunomodulatory and antibacterial activity against multidrug-resistant *Acinetobacter baumannii*. Microb. Pathog..

[21] Murata M., Wakabayashi H., Yamauchi K., Abe F. (2013). Identification of milk proteins enhancing the antimicrobial activity of lactoferrin and lactoferricin. J. Dairy Sci..

[22] Chen R., Cole N., Dutta D., Kumar N., Willcox M.D. (2017). Antimicrobial activity of immobilized lactoferrin and lactoferricin. J. Biomed. Mater. Res. Part B Appl. Biomater. J..

[23] Fernandes K.E., Carter D.A. (2017). The antifungal activity of lactoferrin and its derived peptides: Mechanisms of action and synergy with drugs against fungal pathogens. Front. Microbiol..

[24] Sijbrandij T., Ligtenberg A.J., Nazmi K., Veerman E.C., Bolscher J.G., Bikker F.J. (2017). Effects of lactoferrin derived peptides on simulants of biological warfare agents. World J. Microbiol. Biotechnol.

[25] Tanhaeian A., Nazifi N., Ahmadi S.F., Akhlaghi M. (2020). Comparative study of antimicrobial activity between some medicine plants and recombinant Lactoferrin peptide against some pathogens of cultivated button mushroom. Arch. Microbiol..

[26] Striednig B., Hilbi H. (2022). Bacterial quorum sensing and phenotypic heterogeneity: How the collective shapes the individual. Trends Microbiol..

[27] Tian X., Ding H., Ke W., Wang L. (2021). Quorum sensing in fungal species. Annu. Rev. Microbiol..

[28] Sikdar R., Elias M. (2020). Quorum quenching enzymes and their effects on virulence, biofilm, and microbiomes: A review of recent advances. Expert Rev. Anti-Infect. Ther..

[29] Kusada H., Zhang Y., Tamaki H., Kimura N., Kamagata Y. (2019). Novel N-acyl homoserine lactone-degrading bacteria isolated from penicillin-contaminated environments and their quorum-quenching activities. Front. Microbiol..

[30] Rezzoagli C., Archetti M., Mignot I., Baumgartner M., Kümmerli R. (2020). Combining antibiotics with antivirulence compounds can have synergistic effects and reverse selection for antibiotic resistance in *Pseudomonas aeruginosa*. PLoS Biol..

[31] Aslanli A., Domnin M., Stepanov N., Efremenko E. (2022). “Universal” antimicrobial combination of bacitracin and His_6_-OPH with lactonase activity, acting against various bacterial and yeast cells. Int. J. Mol. Sci..

[32] Aslanli A., Lyagin I., Efremenko E. (2019). Charges’ interaction in polyelectrolyte (nano) complexing of His_6_-OPH with peptides: Unpredictable results due to imperfect or useless concept?. Int. J. Biol. Macromol..

[33] Aslanli A., Lyagin I., Stepanov N., Presnov D., Efremenko E. (2020). Bacterial cellulose containing combinations of antimicrobial peptides with various QQ enzymes as a prototype of an “enhanced antibacterial” dressing: In silico and in vitro data. Pharmaceutics.

[34] Efremenko E., Lyagin I., Votchitseva Y., Sirotkina M., Varfolomeyev S. (2007). Polyhistidine-containing organophosphorus hydrolase with outstanding properties. Biocatal. Biotransformation.

[35] Andersen J.H., Osbakk S.A., Vorland L.H., Traavik T., Gutteberg T.J. (2001). Lactoferrin and cyclic lactoferricin inhibit the entry of human cytomegalovirus into human fibroblasts. Antivir. Res..

[36] Huertas Mendez N.D.J., Vargas Casanova Y., Gomez Chimbi A.K., Hernández E., Leal Castro A.L., Melo Diaz J.M., Rivera Monroy Z.J., Garcia Castaneda J.E. (2017). Synthetic peptides derived from bovine lactoferricin exhibit antimicrobial activity against *E. coli* ATCC 11775, *S. maltophilia* ATCC 13636 and *S. enteritidis* ATCC 13076. Molecules.

[37] Efremenko E.N., Lyagin I.V., Klyachko N.L., Bronich T., Zavyalova N.V., Jiang Y., Kabanov A.V. (2017). A simple and highly effective catalytic nanozyme scavenger for organophosphorus neurotoxins. J. Control Release.

[38] Lyagin I., Stepanov N., Maslova O., Senko O., Aslanli A., Efremenko E. (2022). Not a mistake but a feature: Promiscuous activity of enzymes meeting mycotoxins. Catalysts.

[39] Lyagin I.V., Efremenko E.N. (2018). Biomolecular engineering of biocatalysts hydrolyzing neurotoxic organophosphates. Biochimie.

[40] Artym J., Zimecki M. (2021). Antimicrobial and Prebiotic Activity of Lactoferrin in the Female Reproductive Tract: A Comprehensive Review. Biomedicines.

[41] Efremenko E.N., Ugarova N.N., Lomakina G.Y., Senko O.V., Stepanov N.A., Maslova O.V., Aslanly A.G., Lyagin I.V. (2022). Bioluminescent ATP-Metry: Practical Aspects.

[42] Jorge P., Alves D., Pereira M.O. (2019). Catalysing the way towards antimicrobial effectiveness: A systematic analysis and a new online resource for antimicrobial–enzyme combinations against *Pseudomonas aeruginosa* and *Staphylococcus aureus*. Int. J. Antimicrob. Agents.

[43] Laulund A.S., Schwartz F.A., Christophersen L., Høiby N., Svendsen J.S.M., Stensen W., Thomsen K., Cavanagh J.P., Moser C. (2022). Lactoferricin-inspired peptide AMC-109 augments the effect of ciprofloxacin against Pseudomonas aeruginosa biofilm in chronic murine wounds. J. Glob. Antimicrob. Resist..

[44] Intorasoot S., Intorasoot A., Tawteamwong A., Butr-Indr B., Phunpae P., Tharinjaroen C.S., Wattananandkul U., Sangboonruang S., Khantipongse J. (2022). In vitro antimycobacterial activity of human lactoferrin-derived peptide, d-hlf 1-11, against susceptible and drug-resistant *Mycobacterium tuberculosis* and its synergistic effect with rifampicin. Antibiotics.

[45] Ibarra-Sánchez L.A., Kong W., Lu T., Miller M.J. (2021). Efficacy of nisin derivatives with improved biochemical characteristics, alone and in combination with endolysin PlyP100 to control *Listeria monocytogenes* in laboratory-scale Queso Fresco. Food Microbiol..

[46] Blumenthal I., Davis L.R., Berman C.M., Griswold K.E. (2021). Nonclassical antagonism between human lysozyme and AMPs against *Pseudomonas aeruginosa*. FEBS Open Bio..

[47] Bruni N., Capucchio M.T., Biasibetti E., Pessione E., Cirrincione S., Giraudo L., Corona A., Dosio F. (2016). Antimicrobial activity of lactoferrin-related peptides and applications in human and veterinary medicine. Molecules.

[48] Djokic L., Stankovic N., Galic I., Moric I., Radakovic N., Šegan S., Pavic A., Senerovic L. (2022). Novel quorum quenching YtnP lactonase from *Bacillus paralicheniformis* reduces *Pseudomonas aeruginosa* virulence and increases antibiotic efficacy *in vivo*. Front. Microbiol..

[49] Aslanli A., Lyagin I., Efremenko E. (2018). Novel approach to Quorum Quenching: Rational design of antibacterials in combination with hexahistidine-tagged organophosphorus hydrolase. Biol. Chem..

[50] Vega-Bautista A., de la Garza M., Carrero J.C., Campos-Rodríguez R., Godínez-Victoria M., Drago-Serrano M.E. (2019). The impact of lactoferrin on the growth of intestinal inhabitant bacteria. Int. J. Mol. Sci..

[51] Chen P.W., Jheng T.T., Shyu C.L., Mao F.C. (2013). Antimicrobial potential for the combination of bovine lactoferrin or its hydrolysate with lactoferrin-resistant probiotics against foodborne pathogens. J. Dairy Sci..

[52] Wu H., Gao Y., Li S., Bao X., Wang J., Zheng N. (2021). Lactoferrin alleviated AFM1-induced apoptosis in intestinal NCM 460 cells through the autophagy pathway. Foods.

[53] Zheng N., Zhang H., Li S., Wang J., Liu J., Ren H., Gao Y. (2018). Lactoferrin inhibits aflatoxin B1-and aflatoxin M1-induced cytotoxicity and DNA damage in Caco-2, HEK, Hep-G2, and SK-N-SH cells. Toxicon.

[54] Lyagin I., Efremenko E. (2019). Enzymes for detoxification of various mycotoxins: Origins and mechanisms of catalytic action. Molecules.

[55] Lyagin I., Maslova O., Stepanov N., Efremenko E. (2022). Degradation of mycotoxins in mixtures by combined proteinous nanobiocatalysts: *In silico*, in vitro and *in vivo*. Int. J. Biol. Macromol..

[56] Errante F., Ledwoń P., Latajka R., Rovero P., Papini A.M. (2020). Cosmeceutical peptides in the framework of sustainable wellness economy. Front. Chem..

[57] Pryshchepa O., Pomastowski P., Rafińska K., Gołębiowski A., Rogowska A., Monedeiro-Milanowski M., Sagandykova G., Michalke B., Schmitt-Kopplin P., Gloc M. (2022). Synthesis, physicochemical characterization, and antibacterial performance of silver—Lactoferrin complexes. Int. J. Mol. Sci..

[58] Manzoni P., Rinaldi M., Cattani S., Pugni L., Romeo M.G., Messner H., Stolfi I., Decembrino L., Laforgia N., Vagnarelli F. (2009). Italian task force for the study and prevention of neonatal fungal infections, italian society of neonatology. bovine lactoferrin supplementation for prevention of late-onset sepsis in very low-birth-weight neonates: A randomized trial. JAMA.

[59] Aguirre-Guataqui K., Márquez-Torres M., Pineda-Castañeda H.M., Vargas-Casanova Y., Ceballos-Garzon A., Rivera-Monroy Z.J., García-Castañeda J.E., Parra-Giraldo C.M. (2022). Chimeric peptides derived from bovine lactoferricin and buforin II: Antifungal activity against reference strains and clinical isolates of *Candida spp*. Antibiotics.

[60] Obozina A.S., Komedchikova E.N., Kolesnikova O.A., Iureva A.M., Kovalenko V.L., Zavalko F.A., Rozhnikova T.V., Tereshina E.D., Mochalova E.N., Shipunova V.O. (2023). Genetically encoded self-assembling protein nanoparticles for the targeted delivery in vitro and *in vivo*. Pharmaceutics.

[61] Bruno F., Malvaso A., Canterini S., Bruni A.C. (2022). Antimicrobial peptides (AMPs) in the pathogenesis of Alzheimer’s disease: Implications for diagnosis and treatment. Antibiotics.

[62] Aslanli A., Lyagin I., Efremenko E. (2022). Decarboxylases as hypothetical targets for actions of organophosphates: Molecular modeling for prediction of hidden and unexpected health threats. Food Chem. Toxicol..

[63] Dolinsky T.J., Czodrowski P., Li H., Nielsen J.E., Jensen J.H., Klebe G., Baker N.A. (2007). PDB2PQR: Expanding and upgrading automated preparation of biomolecular structures for molecular simulations. Nucleic Acids Res..

[64] Morris G.M., Huey R., Lindstrom W., Sanner M.F., Belew R.K., Goodsell D.S., Olson A.J. (2009). AutoDock4 and Auto-DockTools4: Automated docking with selective receptor flexibility. J. Comput. Chem..

[65] Trott O., Olson A.J. (2010). AutoDock Vina: Improving the speed and accuracy of docking with a new scoring function, efficient optimization, and multithreading. J. Comput. Chem..

[66] Tomita M., Bellamy W., Takase M., Yamauchi K., Wakabayashi H., Kawase K. (1991). Potent antibacterial peptides generated by pepsin digestion of bovine lactoferrin. J. Dairy Sci..

[67] Chan J.C., Li-Chan E.C. (2007). Production of lactoferricin and other cationic peptides from food grade bovine lactoferrin with various iron saturation levels. J. Agric. Food Chem..

[68] Gattiker A., Bienvenut W.V., Bairoch A., Gasteiger E. (2002). FindPept, a tool to identify unmatched masses in peptide mass fingerprinting protein identification. Proteomics.

[69] Efremenko E., Votchitseva Y., Plieva F., Galaev I., Mattiasson B. (2006). Purification of His_6_-organophosphate hydrolase using monolithic supermacroporous polyacrylamide cryogels developed for immobilized metal affinity chromatography. Appl. Microbiol. Biotechnol..

[70] Veselov M.M., Uporov I.V., Efremova M.V., Le-Deygen I.M., Prusov A.N., Shchetinin I.V., Savchenko A.G., Golovin Y.I., Kabanov A.V., Klyachko N.L. (2022). Modulation of α-Chymotrypsin Conjugated to Magnetic Nanoparticles by the Non-Heating Low-Frequency Magnetic Field: Molecular Dynamics, Reaction Kinetics, and Spectroscopy Analysis. ACS Omega.

[71] Li H., Robertson A.D., Jensen J.H. (2005). Very fast empirical prediction and rationalization of protein pKa values. Proteins Struct. Funct. Genet..

[72] Baker N.A., Sept D., Joseph S., Holst M.J., McCammon J.A. (2001). Electrostatics of nanosystems: Application to microtubules and the ribosome. Proc. Natl. Acad. Sci. USA.

