# Supplementary material for: Synergistic Antimicrobial Action of Lactoferrin-Derived Peptides and Quorum Quenching Enzymes
Source: Int J Mol Sci. 2023 Feb 10;24(4):3566. doi: 10.3390/ijms24043566 (PMC9965131; doi:10.3390/ijms24043566)
Supplement: Supplementary file 1 [file ijms-24-03566-s001.zip › Supplementary Materials_S1.pdf]

Supplementary Materials

International Journal of Molecular Sciences

Synergistic antimicrobial action of lactoferrin derived peptides and quorum quenching enzymes

Aysel Aslanli, Maksim Domnin, Nikolay Stepanov and Elena Efremenko*

Chemical Faculty, Lomonosov Moscow State University, Lenin Hills 1/3, 119991 Moscow, Russia; ayselaslanli@mail.ru (A.A.); domninmaxchem@gmail.com (M.D.); na.stepanov@gmail.com (N.S.); elena_efremenko@list.ru (E.E.)
Correspondence: elena_efremenko@list.ru; Tel.: +7-495-939-3170; Fax: +7-495-939-5417

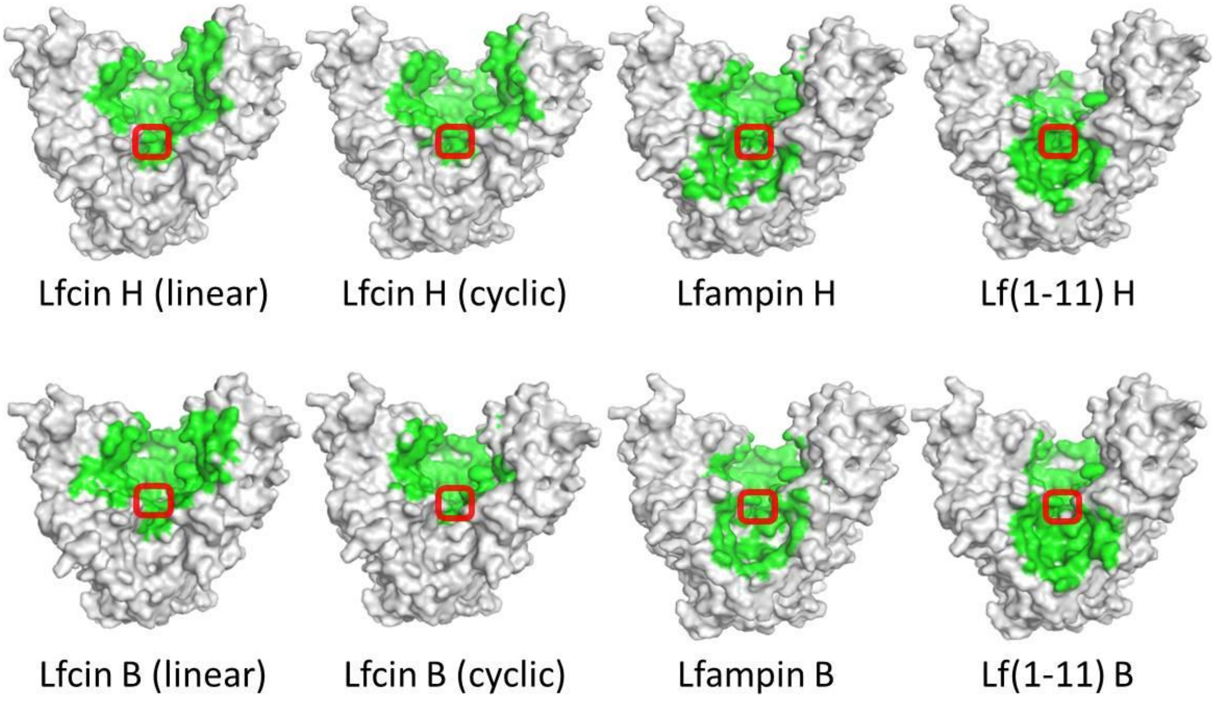

Figure S1. Front view of domains for binding of lactoferrin-derived AMPs at pH 7.5 on the surface of penicillin acylase (PvdQ acylase) colored gray. The atoms located within 4 Å of any AMP atom and the corresponding molecular surface, are colored green. The entrances to the active sites of PvdQ acylase are highlighted with red boxes.

Table S1. Total surface charge of AMPs

AMP	Charge at pH 7.5	Charge at pH 10.5
Lfcin H	7.6	3.1
Lfcin B	8.5	4.3
Lfampin H	- 0.4	- 1.0
Lfampin B	0.6	-1.2
Lf(1–11) H	4.0	4.0
Lf(1–11) B	4.0	2.7

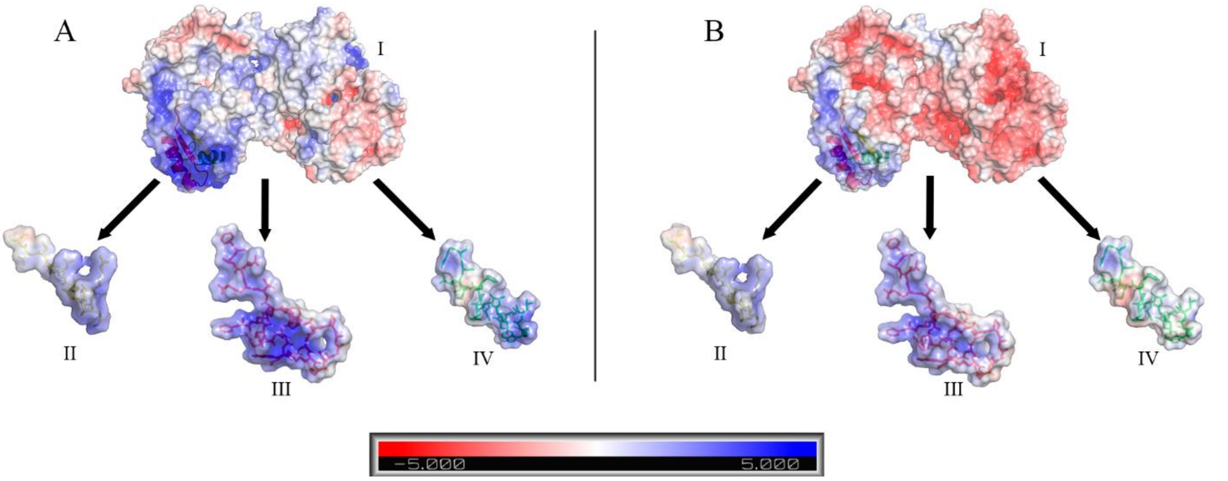

Figure S2. Charge distribution on the surface of bovine Lactoferrin (**I**) and its AMPs Lf(1-11) (**II**), Lactoferricin (**III**) and Lactoferampin (**IV**) at pH 7.5 (**A**) and pH 10.5 (**B**). The red and blue colors mark the negatively and positively charged amino acid groups, respectively. The charge distribution was determined using the 3D crystal structure of the bovine Lactoferrin (Protein Data Base (PDB) code 1BLF). PDB structure was converted to PQR format in PDB2PQR Server for continuum electrostatics calculations using PARSE force field [1]. Additionally the hydrogen bonding network was optimized and protonation state was assigned to pH 7.5 or 10.5 using PropKa [2]. Continuum electrostatic surface of OPH-dimer was solved using Adaptive Poisson-Boltzmann Solver (APBS, ver. 1.3) [3]. The resulting electrostatic surface was visualized in PyMol (ver. 12.0.28).

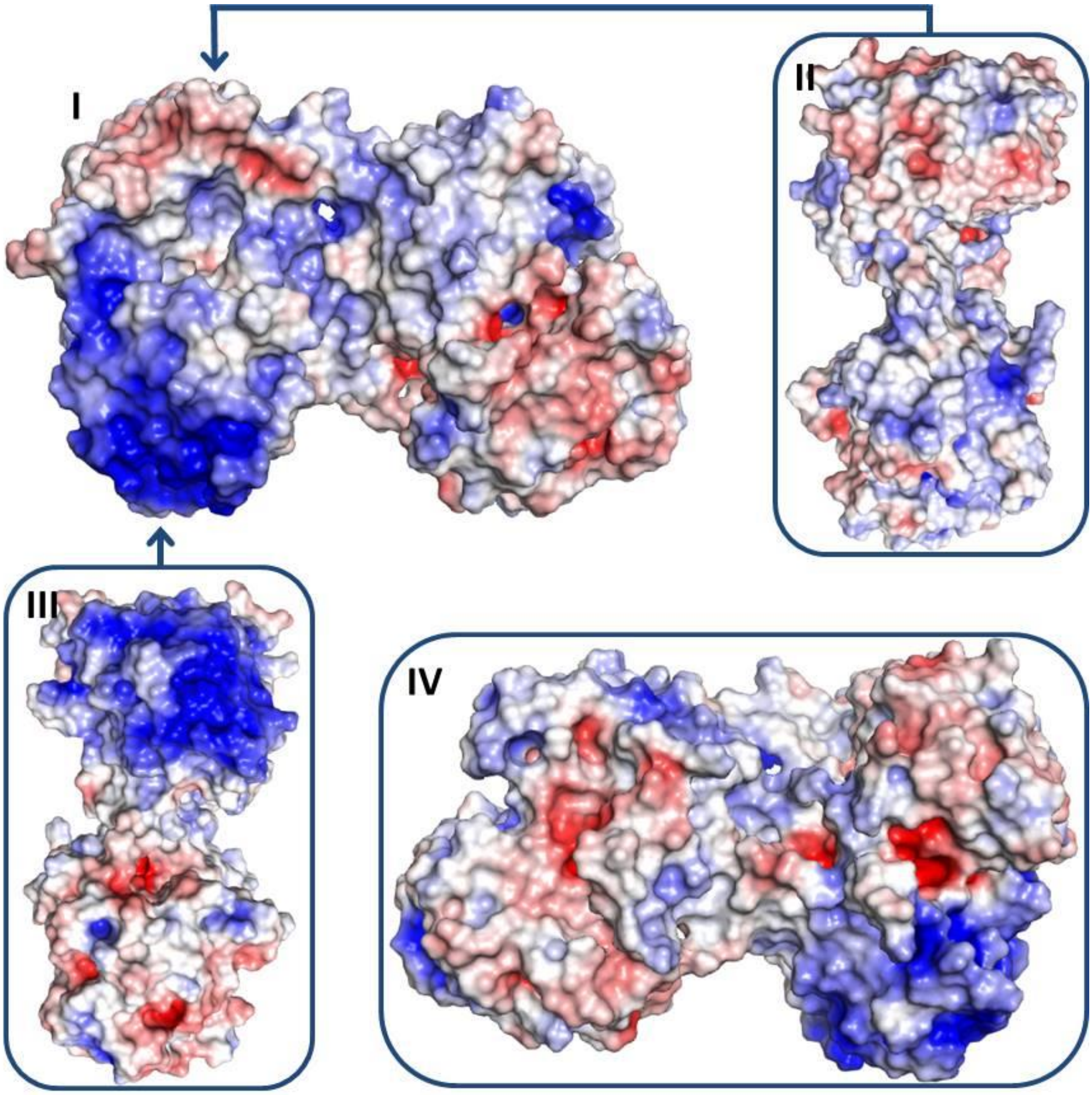

Figure S3. Front view (**I**), top view (**II**). bottom view (**III**) and back view (**IV**) of charge distribution on the surface of bovine Lactoferrin at pH 7.5 (**A**). The red and blue colors mark the negatively and positively charged amino acid groups, respectively. The charge distribution was determined using the 3D crystal structure of the bovine Lactoferrin (Protein Data Base (PDB) code 1BLF). PDB structure was converted to PQR format in PDB2PQR Server for continuum electrostatics calculations using PARSE force field [1]. Additionally the hydrogen bonding network was optimized and protonation state was assigned to pH 7.5 or 10.5 using PropKa [2]. Continuum electrostatic surface of OPH-dimer was solved using Adaptive Poisson-Boltzmann Solver (APBS, ver. 1.3) [3]. The resulting electrostatic surface was visualized in PyMol (ver. 12.0.28).

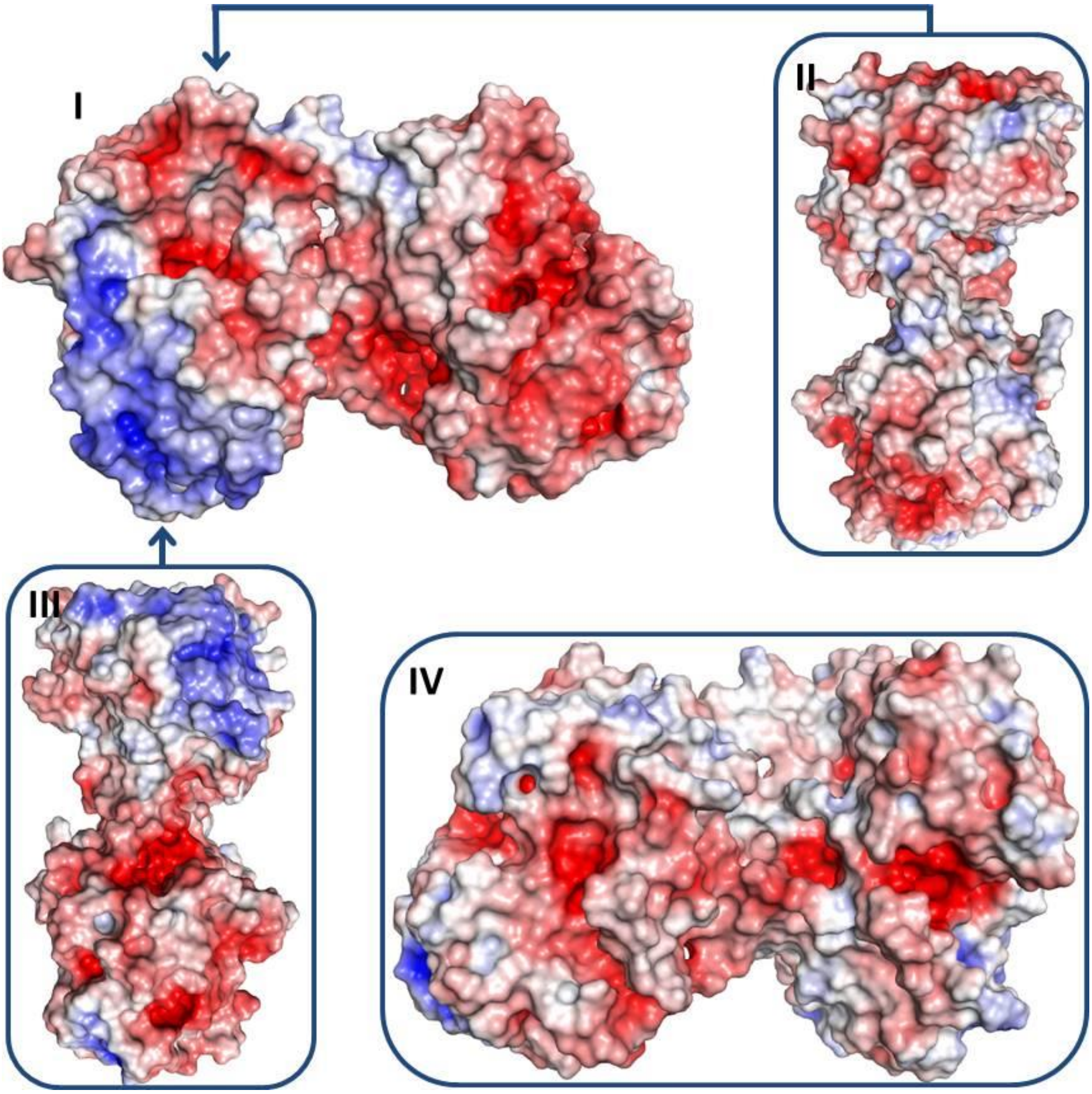

Figure S4. Front view (**I**), top view (**II**). bottom view (**III**) and back view (**IV**) of charge distribution on the surface of bovine Lactoferrin at pH 7.5 (**A**). The red and blue colors mark the negatively and positively charged amino acid groups, respectively. The charge distribution was determined using the 3D crystal structure of the bovine Lactoferrin (Protein Data Base (PDB) code 1BLF). PDB structure was converted to PQR format in PDB2PQR Server for continuum electrostatics calculations using PARSE force field [1]. Additionally the hydrogen bonding network was optimized and protonation state was assigned to pH 7.5 or 10.5 using PropKa [2]. Continuum electrostatic surface of OPH-dimer was solved using Adaptive Poisson-Boltzmann Solver (APBS, ver. 1.3) [3]. The resulting electrostatic surface was visualized in PyMol (ver. 12.0.28).

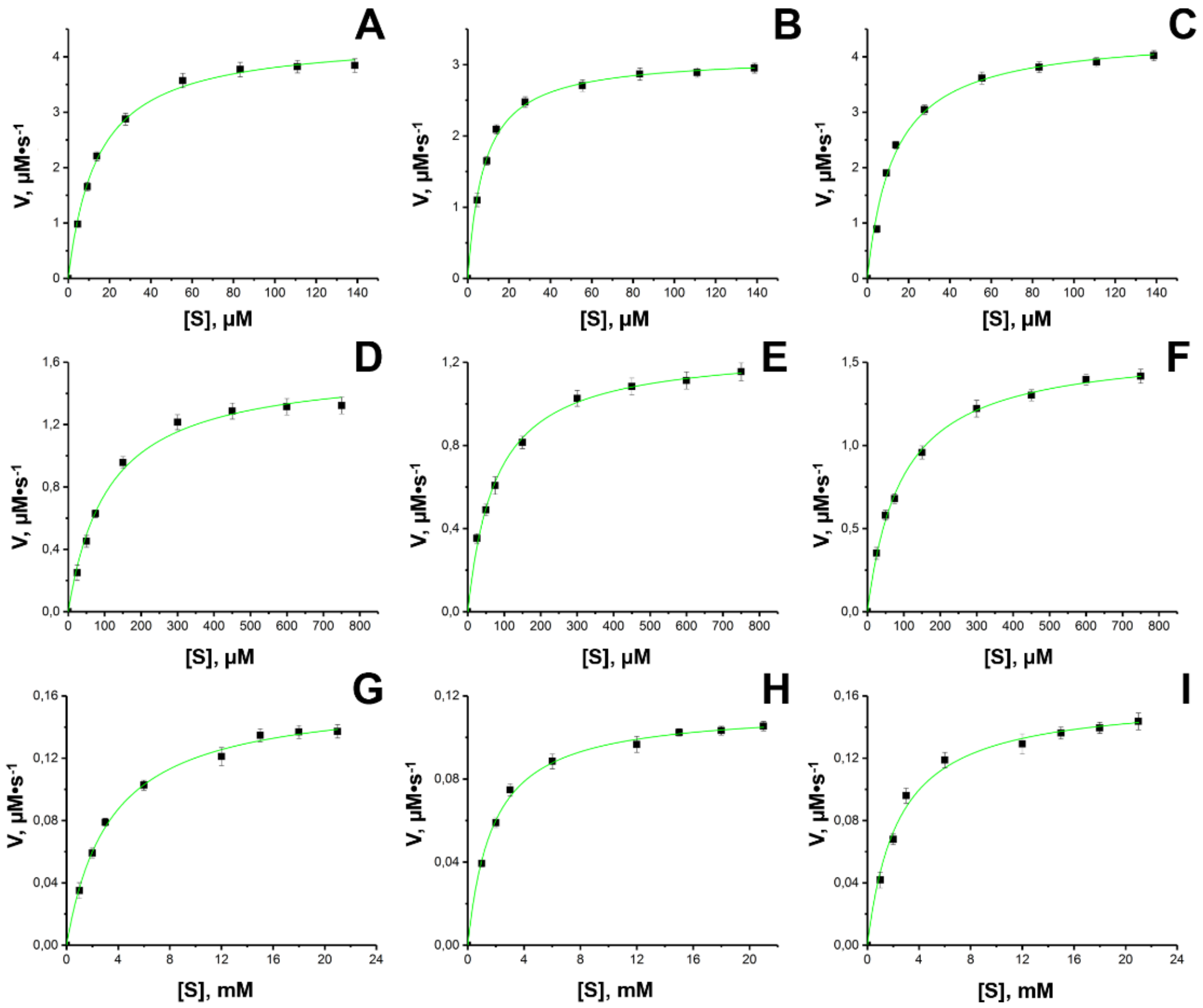

Figure S5. Michaelis-Menten plots of His$_6$-OPH enzyme kinetics in the reactions of hydrolysis of Paraoxon (**A,B,C**); N-Acyl homoserine lactone (**D,E,F**) and mycotoxin (**G,H,I**) molecules alone (**A,D,G**) or in combination with Lactoferrin (**B,E,H**) or Lfcin (**C,F,I**).

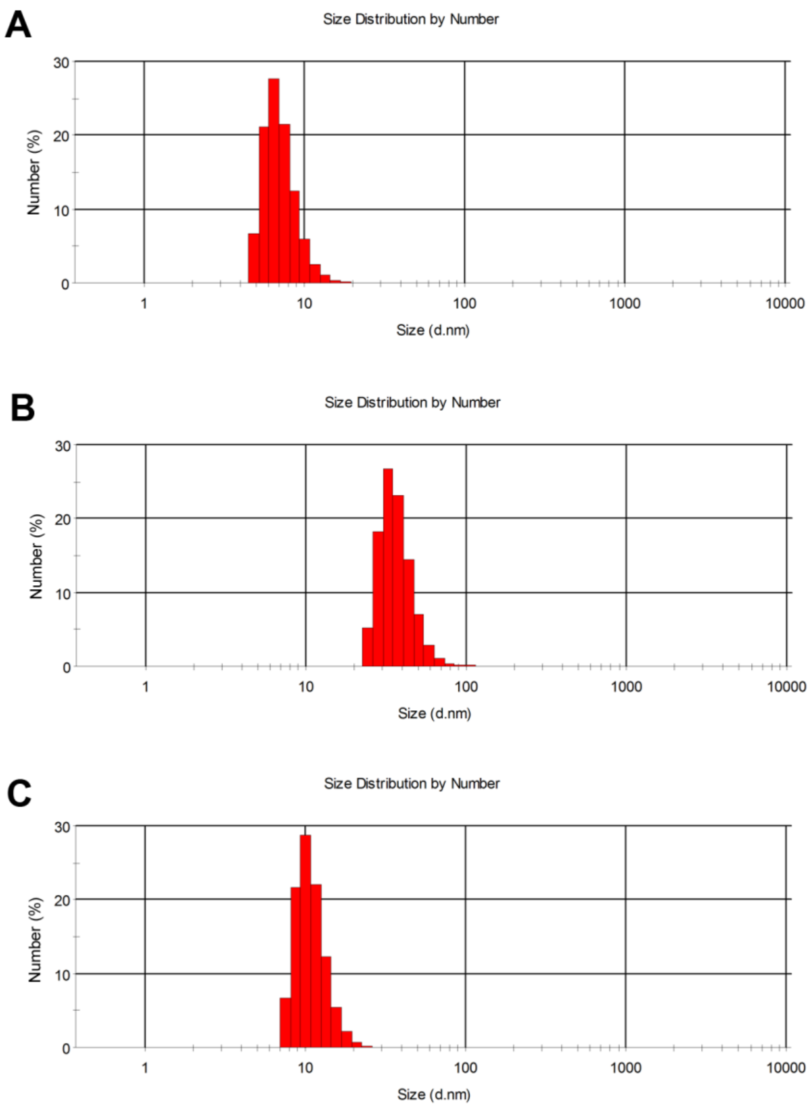

Figure S6. Dynamic light scattering (DLS) size distribution by number of particle of His_6-OPH (**A**) and its combinations with bovine Lactoferrin (**B**) and Lactoferricin (**C**). Samples were prepared at pH 7.5. Protein concentration in the samples was 0.2 g/L in 50 mM phosphate buffer (pH 7.5) containing 150 mM NaCl. Effective hydrodynamic diameters (Deff) were determined using a Zetasizer Nano ZS (Malvern Instruments Ltd., UK). All measurements were performed in automatic mode at 25^0C. Software provided by the manufacturer was used to convert the intensity distribution to the number distribution. All measurements were performed at least in triplicate.

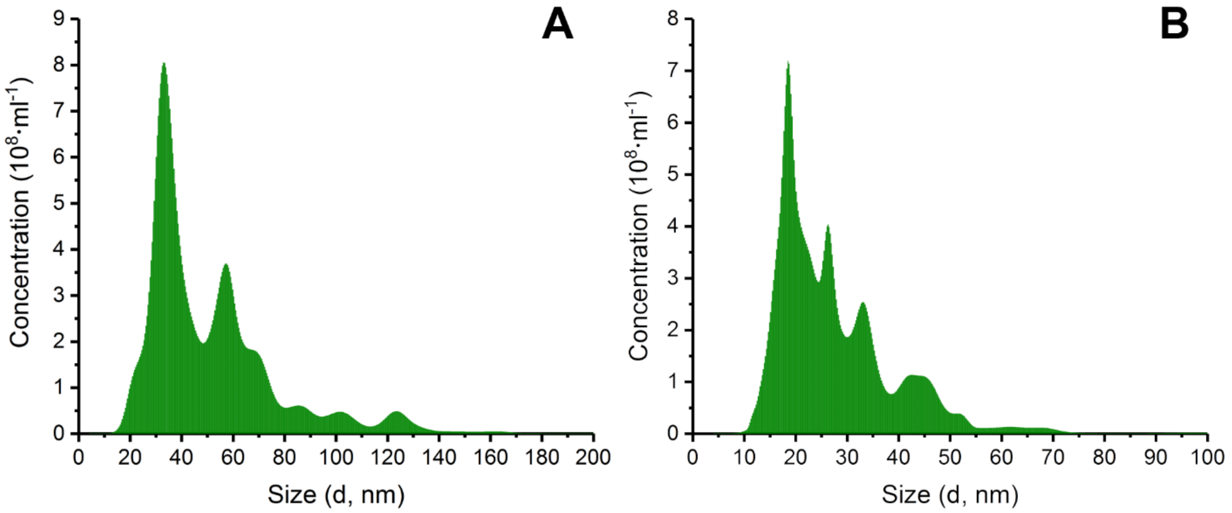

Figure S7. Nanoparticle tracking analysis of combinations of His_6-OPH with Lactoferrin (**A**) and Lactoferricin (**B**).

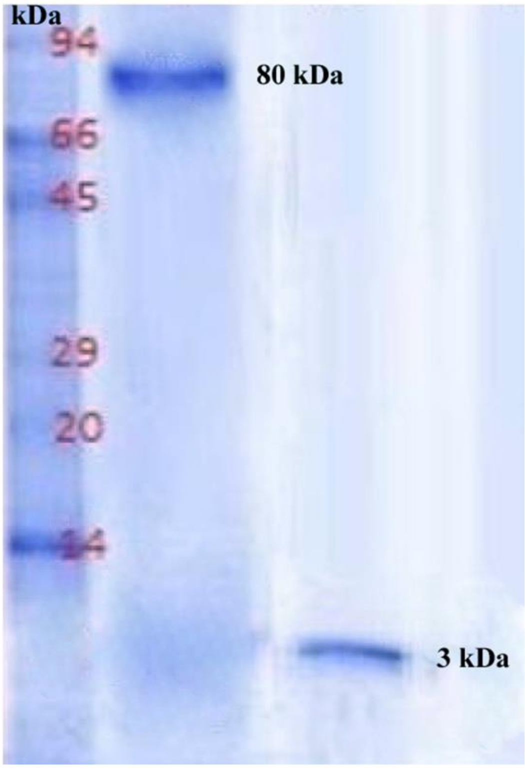

Figure S8. Electrophoregram of bovine lactoferrin (**80 kDa**) and fraction of purified Lfcin (**3 kDa**).

References

1. Dolinsky, T.J.; Czodrowski, P.; Li, H.; Nielsen, J.E.; Jensen, J.H.; Klebe, G.; Baker, N.A. PDB2PQR: Expanding and upgrading automated preparation of biomolecular structures for molecular simulations. *Nucleic Acids Res*. **2007**, *35*, W522–W525. doi:10.1093/nar/gkm276.
2. Li, H.; Robertson, A.D.; Jensen, J.H. Very fast empirical prediction and rationalization of protein pKa values. *Proteins: Struct. Funct. Genet*. **2005**, *61*, 704–721. doi:10.1002/prot.20660.
3. Baker, N.A.; Sept, D.; Joseph, S.; Holst, M.J.; McCammon, J.A. Electrostatics of nanosystems: Application to microtubules and the ribosome. *Proc. Natl. Acad. Sci. USA* **2001**, *98*, 10037–10041. doi:10.1073/pnas.181342398.
